# Direct observation of multiple rotational stacking faults coexisting in freestanding bilayer MoS_2_

**DOI:** 10.1038/s41598-017-07615-9

**Published:** 2017-08-16

**Authors:** Zuocheng Li, Xingxu Yan, Zhenkun Tang, Ziyang Huo, Guoliang Li, Liying Jiao, Li-Min Liu, Miao Zhang, Jun Luo, Jing Zhu

**Affiliations:** 1 National Center for Electron Microscopy in Beijing, School of Materials Science and Engineering, The State Key Laboratory of New Ceramics and Fine Processing, Key Laboratory of Advanced Materials (MOE), Tsinghua University, Beijing, 100084 China; 2 Beijing Computational Science Research Center, Beijing, 100094 China; 3 College of Physics and Electronics Engineering, Hengyang Normal University, Hengyang, 421008 China; 4 Queensland Micro- and Nano Centre, Griffith University, Brisbane, 4111 Australia; 5 Center for Electron Microscopy, TUT-FEI Joint Laboratory, Institute for New Energy Materials & Low-Carbon Technologies, School of Materials Science and Engineering, Tianjin University of Technology, Tianjin, 300384 China; 6 Key Laboratory of Organic Optoelectronics & Molecular Engineering, Department of Chemistry, Tsinghua University, Beijing, 100084 China; 7 Chemical Sciences Division, Lawrence Berkeley National Laboratory, Berkeley, California 94720 USA

## Abstract

Electronic properties of two-dimensional (2D) MoS_2_ semiconductors can be modulated by introducing specific defects. One important type of defect in 2D layered materials is known as rotational stacking fault (RSF), but the coexistence of multiple RSFs with different rotational angles was not directly observed in freestanding 2D MoS_2_ before. In this report, we demonstrate the coexistence of three RSFs with three different rotational angles in a freestanding bilayer MoS_2_ sheet as directly observed using an aberration-corrected transmission electron microscope (TEM). Our analyses show that these RSFs originate from cracks and dislocations within the bilayer MoS_2_. First-principles calculations indicate that RSFs with different rotational angles change the electronic structures of bilayer MoS_2_ and produce two new symmetries in their bandgaps and offset crystal momentums. Therefore, employing RSFs and their coexistence is a promising route in defect engineering of MoS_2_ to fabricate suitable devices for electronics, optoelectronics, and energy conversion.

## Introduction

Single- and few-layer molybdenum disulfide (MoS_2_) sheets are two-dimensional (2D) semiconductors with nonzero intrinsic bandgaps^1–22^, in contrast to the zero-bandgap graphene^23–33^. By having a nonzero bandgap, 2D MoS_2_ is more suitable as a semiconducting channel for electronics^1–5, 7–12, 14–16, 19, 22^, optoelectronics^1, 6, 7, 11, 16^, spintronics^1, 16^, energy conversion^9, 17^, piezotronics^9^, and photonics^6, 7, 18, 21^, compared to graphene. Significantly, crystal defects easily occur in 2D MoS_2_, whose electronic structures can be modulated using many defects such as dislocations^3, 4, 19^, grain boundaries^3, 4, 19^, point vacancies^4–6^, antisite defects^4, 5^, edges^4^, and dopants^12–14^. Hence, the properties of 2D MoS_2_ can be tailored via defect engineering to develop various functional devices^3–6, 12–14, 19^.

Rotational stacking faults (RSFs) are a type of fundamental defects that are widely present in layered materials such as overlapped or folded single- and few-layer 2D MoS_2_^19–22, 34, 35^, few-layer graphene^27–29^, and bulk MoS_2_^36^. These RSFs deviate from the standard stacking modes of layered structures, such as the AB stacking of few-layer and bulk MoS_2_^1, 20, 36^, and give rise to Moiré patterns as their apparent feature^20–22, 27–29, 34–37^. The changes in the stacking sequences alter the electronic structures of layered materials^27, 28, 34–37^. However, the coexistence of multiple RSFs with different rotational angles have yet to be directly observed in freestanding 2D MoS_2_, although it can bring mixed modulation on the electronic structures of the 2D MoS_2_ free of effects of substrates.

In this contribution we report the first direct observation of three RSFs with different rotational angles coexisting in a freestanding bilayer MoS_2_ sheet, the thinnest 2D MoS_2_ crystal in which RSFs can exist. These RSFs were observed using an aberration-corrected (AC) transmission electron microscope (TEM) with a low accelerating voltage of 80 kV for its electron beam. Their atomic structures were directly imaged and then compared with high-resolution TEM (HRTEM) simulations, which indicated that they originated from a crack and two dislocations in one component layer of the bilayer sheet. Their rotational angles were resolved as 27.80°, 20.27° and 14.60°, respectively. Furthermore, first-principles calculations were utilized to examine their electronic structures and those of RSFs with other rotational angles. The calculations indicate that two RSFs possess nearly symmetrical bandgaps and purely symmetrical offset crystal momentums when their rotational angles are symmetrical about 30°. That is, *E*_g_ (*x*) ≈ *E*_g_ (60° − *x*) and *p* (*x*) = *p* (60° − *x*), where *E*_g_, *x* and *p* denote the bandgap, rotational angle and offset crystal momentum of RSF, respectively. The two symmetries can be utilized to engineer the electronic structure of 2D MoS_2_, and the coexistence of different RSFs can bring different electronic structures into a MoS_2_ sheet. This work introduces new opportunities to develop electronic, optoelectronic and energy-conversion devices from 2D MoS_2_.

## Results and Discussion

The bilayer MoS_2_ sheets in this work were synthesized by annealing MoO_2_ microplates in sulfur vapor and then transferred onto TEM grids with poly (methyl methacrylate)-mediated nanotransfer printing. These procedures are exactly identical to those reported^2^. The AC-TEM characterization for HRTEM was performed by FEI Titan 80–300 with a spherical aberration (*Cs*) corrector for the objective lens at the accelerating voltage of 80 kV. The spherical aberration was set to be negative, which can give high contrast and low noise^23, 25–32, 38–40^ (see more details in the Methods section of Supplementary information).

**Figure 1:**
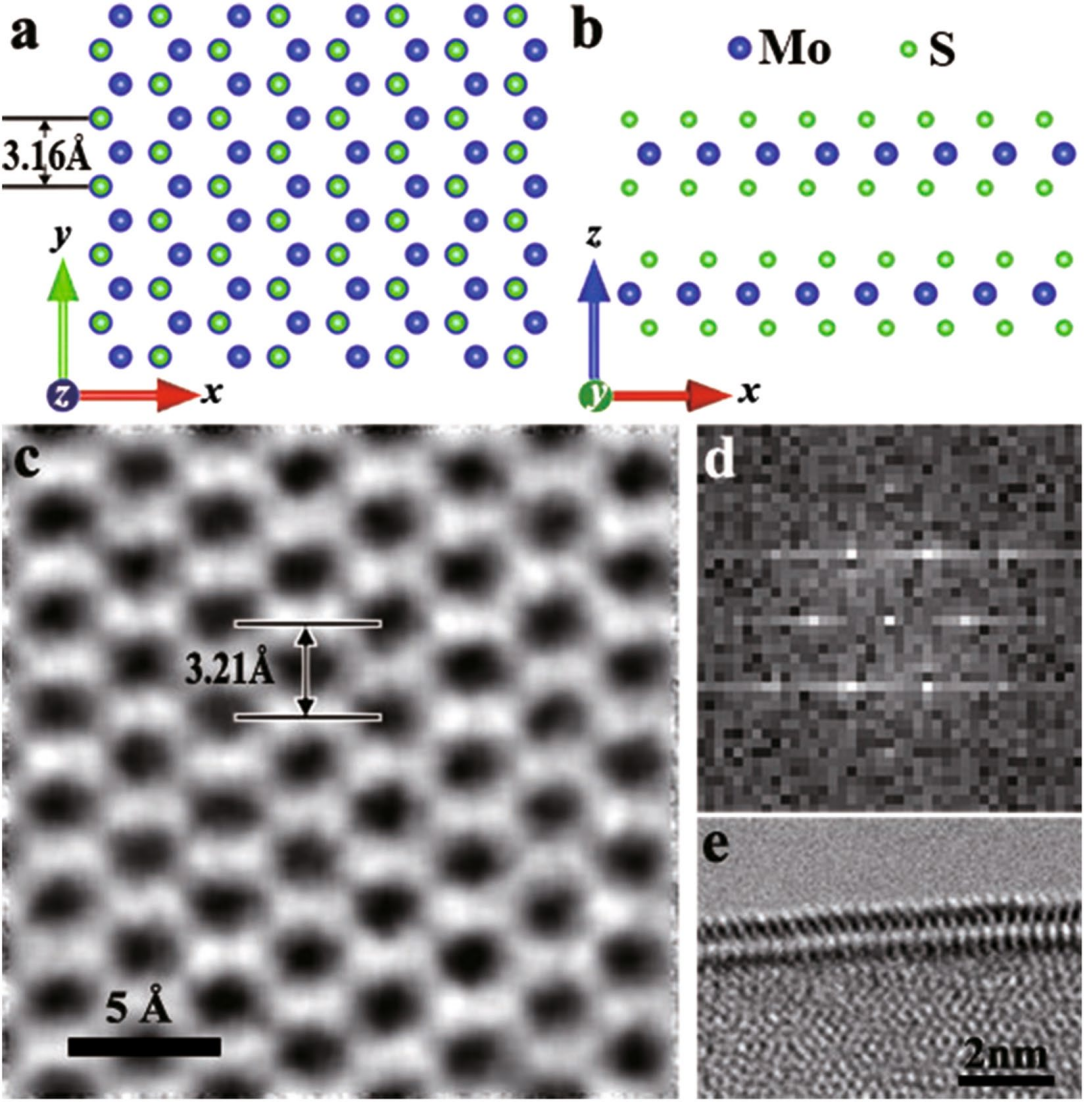
Atomic structure of bilayer MoS_2_ with the standard AB stacking and without any RSFs. (**a,b**) Top and side views of the atomic model of the AB-stacked bilayer MoS_2_. (**c,d**) HRTEM image and its FFT pattern of an AB-stacked bilayer MoS_2_ sheet. This HRTEM image is low-pass filtered and no artifacts are caused by the filtering, which has been widely used to remove noise in HRTEM images^23–32^ (see the unprocessed HRTEM image and more details in Supplementary Fig. S1). The inner six spots adjacent to the center in (**d**) are $\{1\bar{1}00\}$, whereas the outer six spots are $\{1\bar{2}10\}$. (**e**) TEM image of a folded edge of the MoS_2_ sheet. This edge is far away from the area in (**c**).

Figure 1a,b illustrates the atomic model of a bilayer MoS_2_ sheet with the standard AB stacking and without any RSFs. The atomic structure of the theoretical model (Fig. 1a) is a good match to the corresponding HRTEM image (Fig. 1c), with a difference of only 5 pm in the lattice spacing, well within the AC-TEM measurement error. This is a suitable reference for the subsequent analysis of MoS_2_ sheets with RSFs. It is worth noting that no Moiré pattern is observed in the HRTEM image of the freestanding bilayer MoS_2_. The periodic or quasi-periodic spacing of a Moiré pattern is distinctly different from the atomic spacing of the AB-stacked MoS_2_^19–22, 24, 27–29, 34–37^. Figure 1d shows that the six $\{1\bar{1}00\}$ spots in the fast Fourier transform (FFT) pattern of the AB-stacked image constitute only one hexagon, further indicative of the absence of RSFs. Figure 1e shows that the synthesized MoS_2_ sheets are indeed bilayer, as confirmed by the presence of two parallel lines in the TEM image of their folded edges.

**Figure 2:**
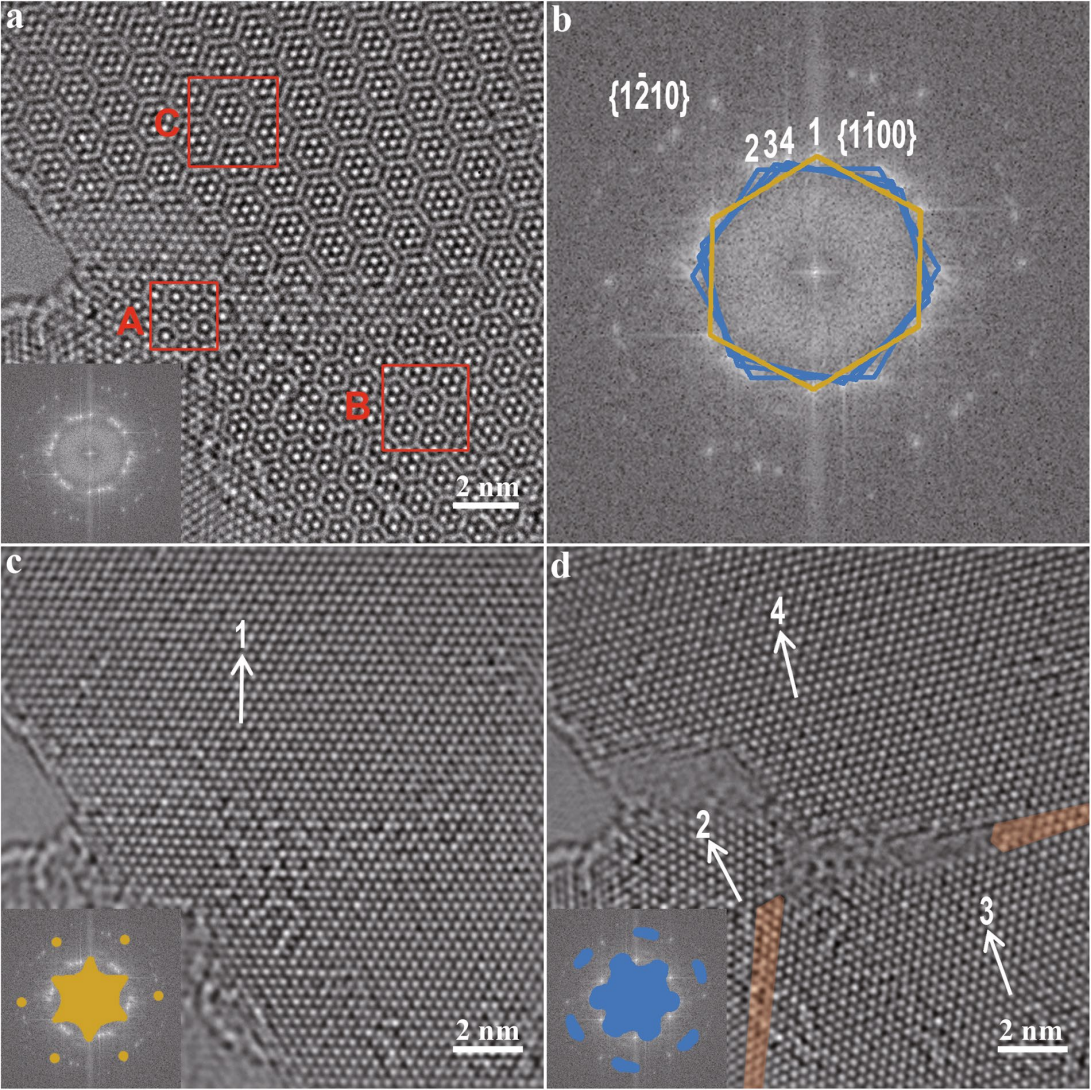
HRTEM images and FFT patterns of a freestanding bilayer MoS_2_ sheet with three RSFs. (**a**) HRTEM image of the sheet containing three Moiré patterns, whose typical zones are marked with the red boxes of A, B and C. This HRTEM image is raw and unfiltered, and the inset is its FFT pattern. (**b**) Enlargement of the FFT pattern in (**a**), where the twenty-four $\{1\bar{1}00\}$ spots constitute four hexagons that are annotated with 1 to 4. Hexagon 1 is highlighted in yellow and the others are in blue. (**c**) HRTEM image reconstruction by the inverse FFT (IFFT) of the information covered by the yellow mask in the inset. The inset is the same image as in (**b**) except that a mask has been applied to exclude Hexagons 2 to 4 and their associated $\{1\bar{2}10\}$ spots. (**d**) HRTEM image reconstruction by the IFFT of the information covered by the blue mask in the inset. The blue mask excludes Hexagon 1 and its associated $\{1\bar{2}10\}$ spots. The semitransparent orange masks in the main panel of (**d**) highlight two dislocations. The arrows in (**c**,**d**) indicate the $[1\bar{1}00]$ orientations corresponding to Hexagons 1 to 4, respectively.

Defects can be produced in intact MoS_2_ sheets by irradiating the sheets with the electron beam of TEM for a long time^4^. We performed the irradiation on freestanding AB-stacked bilayer MoS_2_ sheets and found that Moiré patterns occurred in them (see the Methods section of the Supplementary Information for details of the preparation of the Moiré patterns). Figure 2a shows that an irradiated bilayer MoS_2_ sheet contains three different Moiré patterns. The FFT pattern of the HRTEM image of the sheet contains twenty-four $\{1\bar{1}00\}$ spots, which constitute four hexagons (labelled Hexagons 1 to 4), as illustrated in Fig. 2b. In contrast to Fig. 1c,d, the appearance of the three Moiré patterns with the four hexagons implies that relative rotations and thus three RSFs occur in the two component layers of the bilayer sheet.

Figure 2c gives a reconstructed HRTEM image corresponding to only Hexagon 1, showing a MoS_2_ layer without any cracks. Comparing Fig. 2a and Fig. 2c indicates that the domains of the three Moiré patterns are all contained within the uncracked layer. This means that the uncracked layer is one of the two component layers of the bilayer MoS_2_ sheet with the three Moiré patterns. Since this uncracked layer is exclusively due to Hexagon 1, the HRTEM image of the second component layer of the bilayer MoS_2_ sheet can be reconstructed from Hexagons 2 to 4 (Fig. 2d). This layer is cracked and contains two dislocations, as indicated by the two semitransparent masks in Fig. 2d, of which one is at the crack tip and the other is adjacent to the middle of the crack. The two dislocations are similar to those reported in single-layer MoS_2_ (refs 3, 4, 19) and graphene^23, 25, 26, 31^.

The dislocations and the crack together perturb the in-plane crystallographic orientation of the cracked layer, and divide the layer into three regions with different orientations relative to the uncracked layer. The three regions correspond to Hexagons 2 to 4, respectively, as labelled in Fig. 2d. By comparing their $[1\bar{1}00]$ orientations in Fig. 2c and Fig. 2d, their three rotational angles are given as 27.80°, 20.27° and 14.60°, corresponding to Zones A, B and C, respectively. In addition, the RSFs in Fig. 2 formed instantaneously after the same MoS_2_ region was irradiated by the electron beam of TEM for 317 seconds. This forming was so fast that we did not find opportunities to record any transition states. Lower doses of the electron beam can be tried to control the forming and thus the RSF angles.

**Figure 3:**
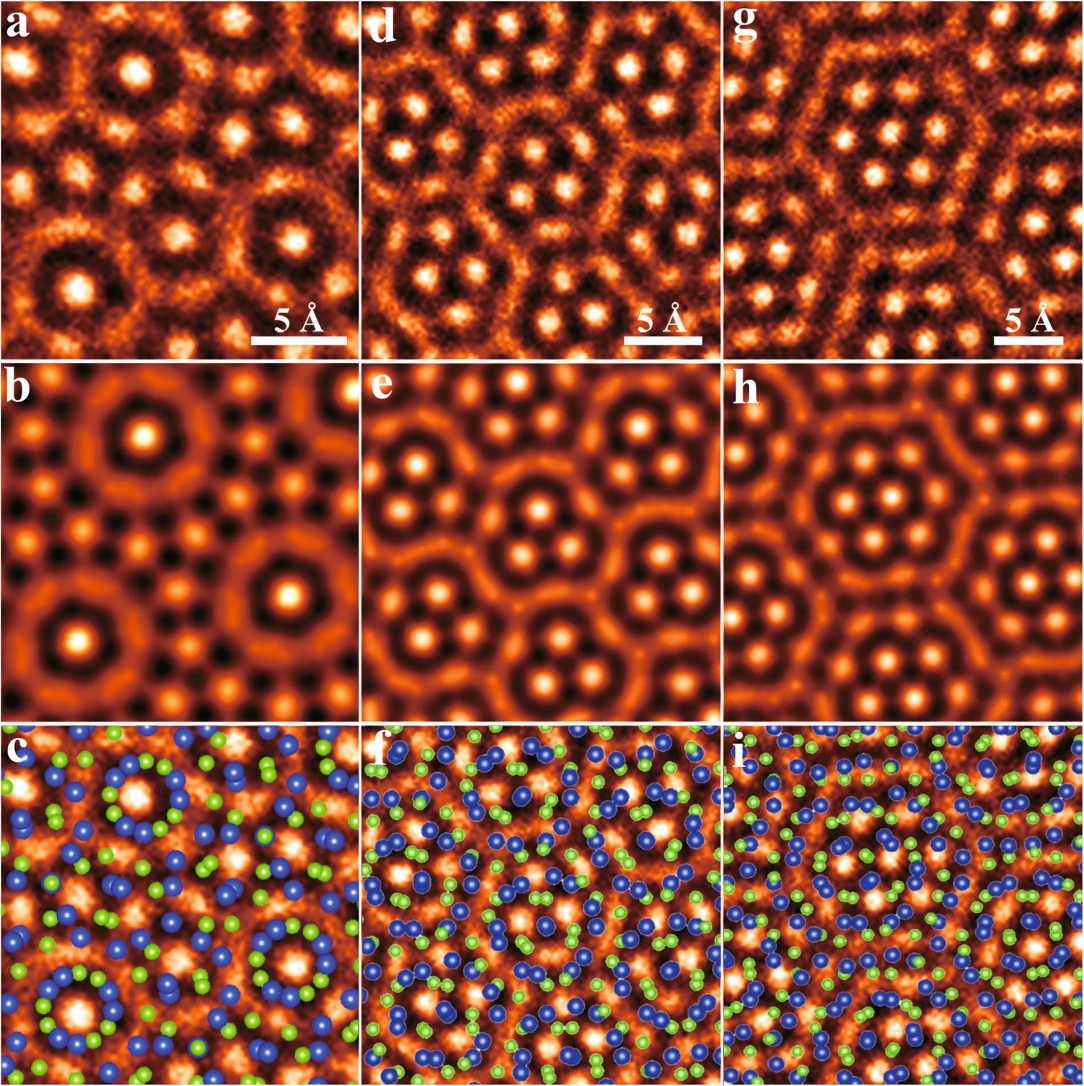
Experimental and simulated HRTEM images of Zones A-C in Fig. 2a. (**a**) Experimental and (**b**) simulated image of Zone A, where false color is used to aid visual inspection. (**c**) Overlay of (**a**) with the atomic model of (**b**), where the blue and the green dots denote the Mo and S atoms, respectively. (**d–f**) and (**g–i**) have the same meanings as (**a–c**) and correspond to Zones B and C, respectively.

Zone A in Fig. 2a corresponds to the rotational angle of 27.80° and is magnified in Fig. 3a. Its atomic model was constructed by adding a rotational angle of 27.80° between two MoS_2_ monolayers (see more details in Supplementary Fig. S2), and then was used to produce a simulated HRTEM image with the MacTempas software^40^ based on the multislice method, which has been widely employed to simulate HRTEM images^25, 27, 28, 30, 32, 38–41^. Figure 3b gives the simulated HRTEM image of Zone A. Figure 3c depicts the overlay of the atomic model of the simulated HRTEM image with the experimentally obtained HRTEM image of Zone A, where areas of bright contrast on the image correspond to voids between the Mo and S atoms. The excellent match between the experimental image (Fig. 3a) and the simulated image as well as its model (Fig. 3b,c) validates the atomic model used. Using a similar methodology but with rotational angles of 20.27° and 14.60° for Zones B and C, respectively, we also show excellent agreement between the experimental HRTEM images with the simulated HRTEM images, as shown in Fig. 3d–i. These results prove that the Moiré patterns observed in the experimental HRTEM images are indeed a superposition between a defect-free monolayer and a monolayer with defects, thereby forming a bilayer of MoS_2_ with multiple RSFs.

To further understand the effects of these RSFs, we performed first-principles calculations to explore their influences on the electronic structure of bilayer MoS_2_ using the Vienna Ab Initio Simulation Package (VASP)^42–47^ (see more details in the Methods section of Supplementary information). In the first-principles calculations, the band structure of a standard MoS_2_ monolayer was firstly tested by the conventional Perdew, Burke and Ernzerhof (PBE) functional^45^ and the hybrid Heyd-Scuseria-Ernzerhof functional (HSE06)^48, 49^ methods, which yielded bandgap values of 1.78 and 2.25 eV, respectively. The value of 1.78 eV is in good agreement with previous calculation results (1.89, 1.78 and 1.9 eV) by PBE^50–52^. Thus, we focused on the PBE band structures in this work. Besides, the influence of spin-orbit coupling in MoS_2_ is reported to be very small, because the Mo and S elements are not heavy enough^53, 54^. Thus, we did not consider the spin-orbit coupling in our calculations.

**Figure 4:**
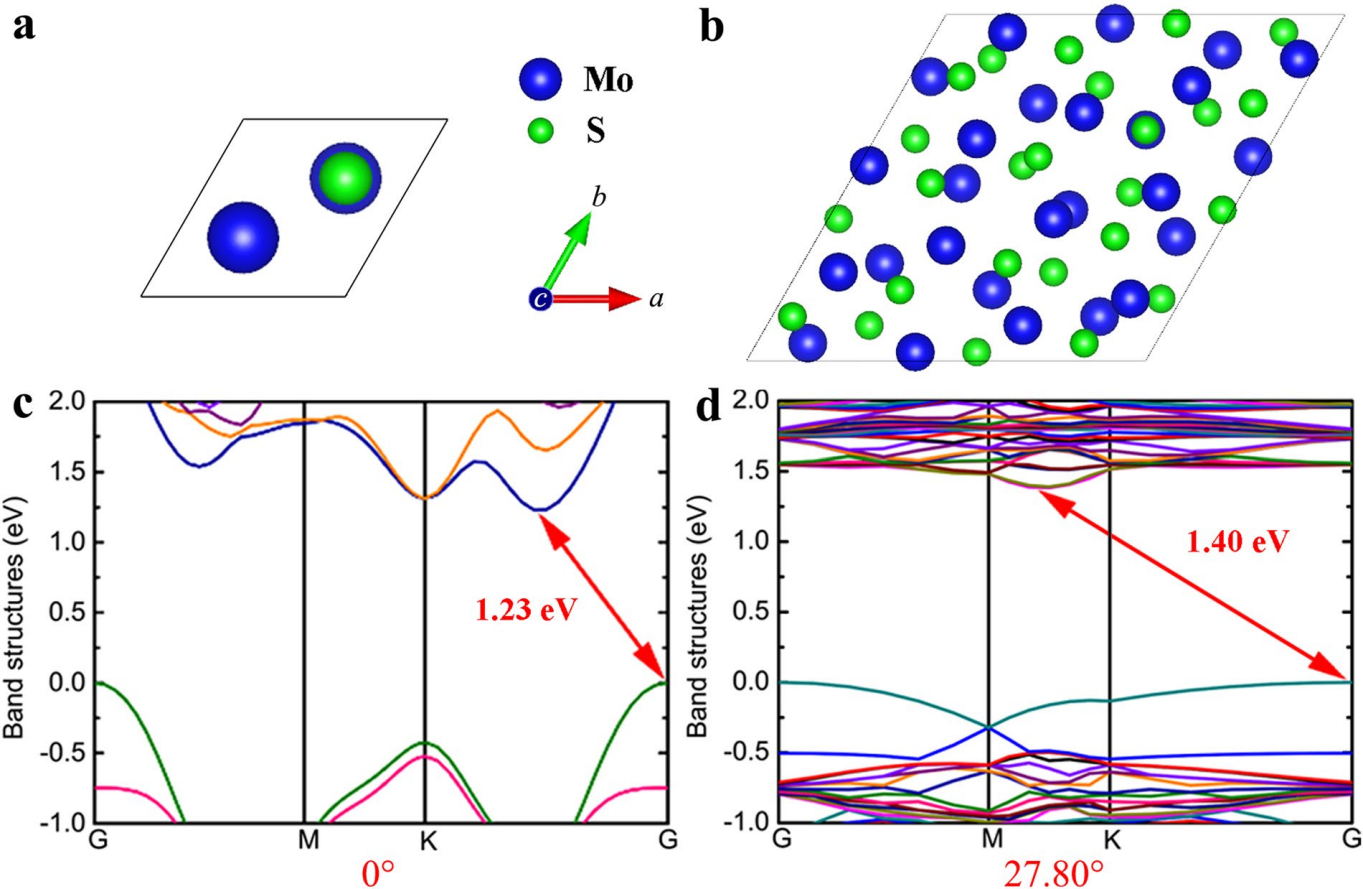
First-principles results of the bilayer MoS_2_ sheets without RSFs and with a RSF of 27.80°, of which the former is standard AB-stacked and can be considered to contain a RSF of 0°. (**a**,**b**) Top views of the unit cells of the two materials, where the *c* direction equals the *z* in Fig. 1a,b. (**c**,**d**) Electronic structures of the two materials obtained by the first-principles calculations, where the two ends of each arrow indicate the positions of CBM and VBM. The red numbers with the unit of eV denote the indirect bandgap values.

Then, we built the unit cells of the AB-stacked bilayer MoS_2_ (Fig. 4a) and a bilayer with the RSF of 27.80° (Fig. 4b). Their corresponding electronic structures were calculated and are depicted in Fig. 4c,d, which indicate that both of them are semiconductors with indirect bandgaps. The bandgap values are calculated to be 1.23 and 1.40 eV, respectively. The valence band maximum (VBM) states of the two materials are located at the G point due to the unchanged interlayer electronic coupling. But, the conduction band minimum (CBM) state of the AB-stacked bilayer MoS_2_ is located between the K and the G points, while the one of the bilayer MoS_2_ containing the 27.80° RSF is between the M and the K points. Thus, the offset crystal momentums of the two materials are 0.774 and 0.409 *h*·nm^−1^, respectively, where *h* is the Planck constant. This difference occurs due to the existence of the RSF rotational angle of 27.80°.

We also tried to calculate the electronic structures of the 20.27° and the 14.60° RSFs, but found that their unit cells contained about 1,300 and 1,480 atoms, respectively, which were too many to be calculated by the computation. Therefore, we approximated those unit cells by building models with the RSFs of 21.78° and 13.17°, which are the calculable models closest to the RSFs of 20.27° and 14.60°. Moreover, the unit cells of the RSFs of 50.57°, 46.83°, 42.10°, 38.22°, 32.20°, 17.90° and 9.43° were also investigated in order to explore the global influence of RSFs on the electronic structure of bilayer MoS_2_. All of the unit cells are displayed in Supplementary Fig. S3, in which the unit cell of the AA-stacked bilayer MoS_2_ was constructed as a reference. It can be considered as a RSF with the rotational angle of 60°.

The electronic structures of all the RSFs of 60°, 50.57°, 46.83°, 42.10°, 38.22°, 32.20°, 21.78°, 17.90°, 13.17° and 9.43° have been calculated and obtained (see Supplementary Fig. S4). They show that regardless of the rotational angle, all of the materials are semiconductors with indirect bandgaps, where all of the VBM states are located at the G point. Their CBM states are found to be between the M and the K points, except for those of the AA- and the AB-stacked, which instead lie between the K and the G points. These results are similar to those of the AB-stacked and the 27.80°. That is, RSFs with rotational angles between 0° and 60° do not move the VBM state but do move the CBM state from between the K and the G points to between the M and the K points. These changes in the electronic structures modify the offset crystal momentum of bilayer MoS_2_. The offset crystal momentum is a key factor in optoelectronics and photovoltaics, where a photon-excited electron in a semiconductor with an indirect bandgap can jump from the valence band to the conduction band only after the offset crystal momentum is transferred between the electron and the crystal lattice. Therefore, a minimal offset crystal momentum is necessary in optoelectronics and photovoltaics with indirect semiconductors, which we can tailor by introducing RSFs of specific angles in the bilayer MoS_2_.

**Figure 5:**
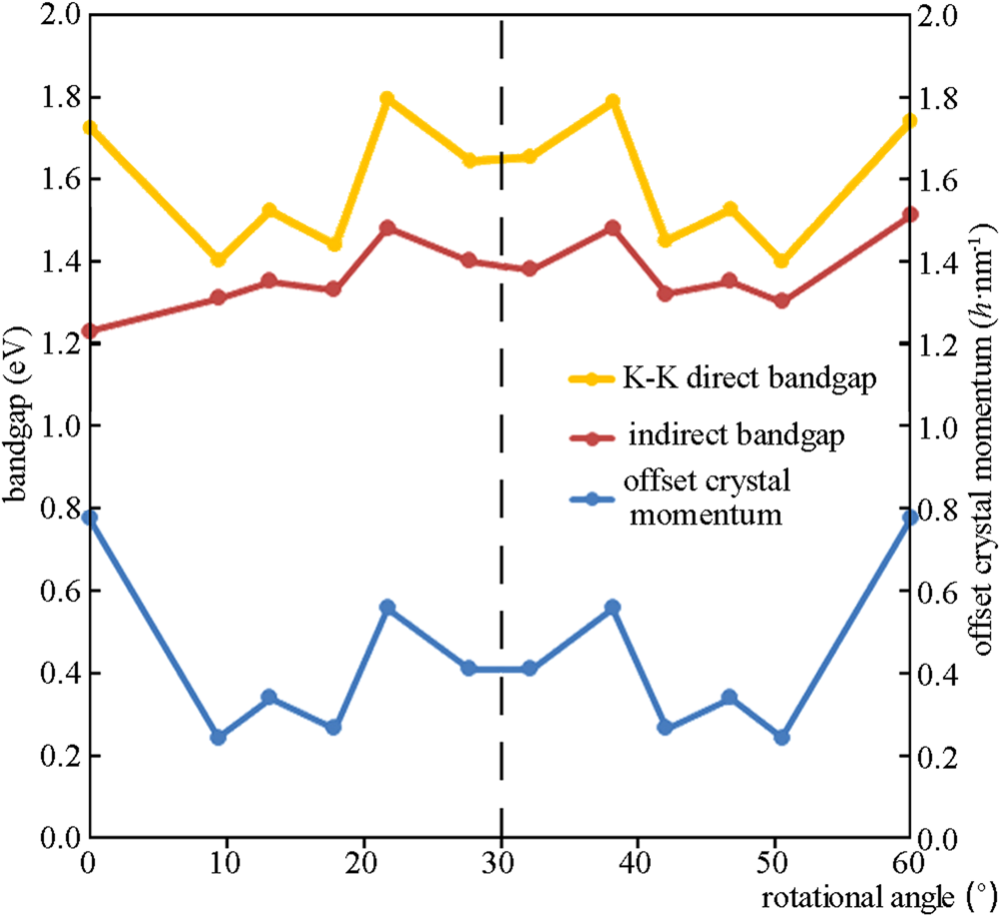
Dependences of the K-K direct bandgap values, the indirect bandgap values and the offset crystal momentums of bilayer MoS_2_ with and without RSFs on the rotational angles. These data are from the calculated electronic structures in Fig. 4c,d and Supplementary Fig. S4.

Figure 5 describes the dependences of the K-K direct bandgap (namely the smallest direct bandgap) values, the indirect bandgap ones and the offset crystal momentums on the rotational angles, indicating that all of the K-K direct bandgap values, the indirect bandgap ones and the offset crystal momentums change with the rotational angles, and they are symmetrical about the rotational angle of 30°. But deviations exist in the symmetry of the bandgaps (including the K-K direct and the indirect), such as the indirect bandgaps of 0° versus 60° and 27.80° versus 32.20°, while no deviations are found in the symmetry of the offset crystal momentums. That is, the bandgaps are nearly symmetrical and the offset crystal momentums are purely symmetrical, which can be written as *E*_g_ (*x*) ≈ *E*_g_ (60° − *x*) and *p* (*x*) = *p* (60° − *x*). *E*_g_, *x* and *p* denote the bandgap, rotational angle and offset crystal momentum of RSF, respectively. The two symmetries were not reported before our work, because some works considered the atoms in the two component layers of RSFs of bilayer MoS_2_ to have nearly random relative distributions^34^ and the others constructed the RSF unit cells with different rotation axes^35^. In our calculation, only the rotational axes passing through the centers of hexagons of the two component layers were used to construct the unit cells (see more details in Supplementary Fig. S3). In particular, the atomic structure of the unit cell with the rotational angle of 27.80° has been used to perform the corresponding HRTEM simulation, as described in Fig. 3b. The good agreement between the experimental and simulated HRTEM images, shown in Fig. 3a–c, validates our consideration of the periodic RSFs. Thus, our calculations are complementary to the reported works^34, 35^. In addition, Fig. 5 shows that the K-K direct bandgap values are comparable to the indirect-gap ones, and the former are 7–40% larger than the latter. The RSF rotational angles modulate not only the indirect bandgap but also the K-K direct bandgap of bilayer MoS_2_.

**Figure 6:**
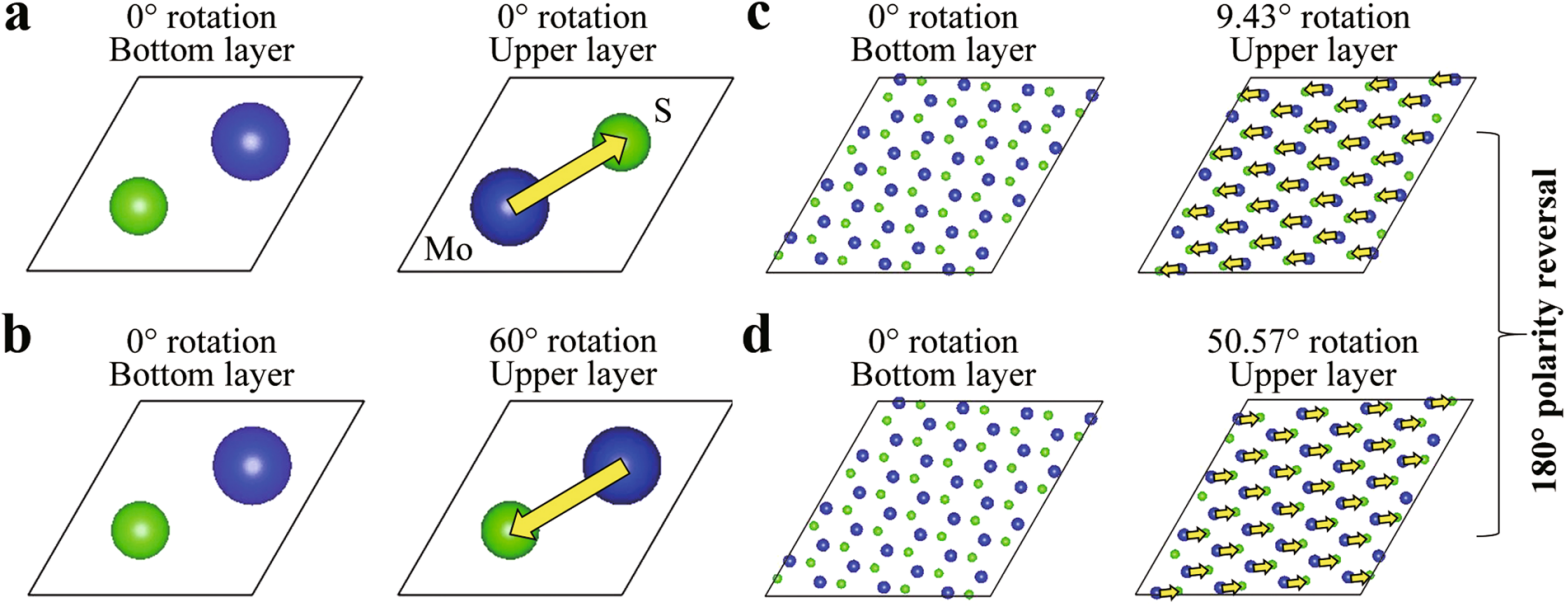
Symmetry between each pair of unit cells with RSFs of the *x* and 60° − *x* rotational angles. (**a**,**b**) Top views of the bottom and upper layers of the AB- and AA-stacked unit cells, whose rotational angles are 0° and 60°, respectively. The yellow arrows highlight the polarity change between the positions of the Mo and S atoms. See more details for the atomic structure of the AA-stacked unit cell in Supplementary Fig. S5. (**c**,**d**) Top views of the bottom and upper layers of the unit cells with RSFs of 9.43° and 50.57°.

The symmetries of the bandgaps and offset crystal momentums occur because the unit cells of two RSFs with two different rotational angels symmetrical about 30° (namely two angles of *x* and 60° − *x*) have similar structures: their sizes and numbers of atoms are identical, and the distributions of the spatial positions of their Mo and S atoms are similar to each other. The similarity of the atomic positions is described as follows: if we leave the bottom MoS_2_ layers immobile while rotating the upper layers by the *x* and 60° − *x* angles respectively, we observe a 180° polarity reversal in the upper layers, such as those shown in Fig. 6. That is, the positions of the atoms of the upper layers rotated by the *x* and 60° − *x* angles are centrosymmetric to each other. This symmetry of the atomic structures consequentially results in the symmetries of the corresponding electronic structures, because atomic structures are the origins of electronic structures^55^. However, bandgaps are also affected by the accurate spatial positions of atoms in atomic structures^55^. Thus, since the atoms of the upper layer of one of the two RSFs in the aforementioned angles have spatial positions that are not exactly the same as but only centrosymmetric to those of the other RSF, the bandgap trend is not purely symmetrical. On the other hand, the fact that a pure symmetry about 30° is observed in the offset crystal momentums suggests that offset crystal momentums are not affected by the accurate spatial positions of atoms but depend on only the symmetry of the atomic structures.

Further, the bandgap of bilayer MoS_2_ with the standard AB stacking is well known to be indirect, meaning that the momentum positions of CBM and VBM in its band structure are different from each other. The difference of the momentum positions is the offset crystal momentum. Our calculations indicate that the band structure of bilayer MoS_2_ is dependent on the rotation angle of RSF, as shown by Fig. 4c,d and Supplementary Fig. S4. It can be seen that RSF makes the band curvatures around CBM and VBM become flatter. This finding can be considered as follows: The unit cells of the MoS_2_ models with RSFs (Fig. 4b and Supplementary Fig. S3b-k) have symmetries different from those of the unit cells of the AB- (Fig. 4a) and the AA-stacked (see Supplementary Fig. S3l). But, the RSF models are all cyclical, implying that their symmetries are not broken but rebuilt. Nevertheless, the sizes of their unit cells are all far bigger than those of the AB- and the AA-stacked, which causes the sampled K-point paths for the RSF models to be concentrated and so leads to the decrease in the curvatures of VBM and CBM.

The flatter curvatures mean that the momentum positions of CBM and VBM can approach each other, that is, the indirect bandgap becomes closer to a direct one. Specifically, Fig. 5 shows that when the rotation angle is 9.43° or 50.57°, the offset crystal momentum value is the smallest found by our calculations. It can be predicted rationally that a rotational angle around 9.43° or 50.57° would be found to make the offset crystal momentum value very close to zero or equal to zero, leading to a quasi-direct or direct bandgap. This search needs a plenty of time for calculations.

**Figure 7:**
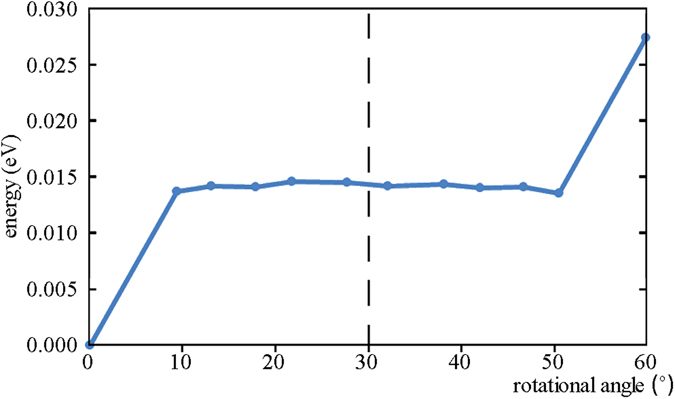
The dependence of the rotational energy per formula unit required to rotate bilayer structure on the RSF rotational angle.

We have calculated the total energies of all the models with the AB stacking, the RSFs and the AA stacking, and then divided them by the formula unit numbers in their corresponding unit cells. The resultant values of energy per formula unit for the models with the RSFs and the AA stacking are different from that of the AB-stacked model, and their differences relative to the latter are the rotational energy per formula unit required to rotate bilayer structure, whose values are plotted in Fig. 7. Increasing temperature can make dislocations in MoS_2_ move^56^. Thus, increasing temperature will affect the rotational angles of RSFs between layers, because the RSF structures are stabilized by dislocations (Fig. 2). It is also indicated that there is an energy barrier to prevent dislocations from moving, and increasing temperature can overcome the barrier^56^. Therefore, there should be an energy barrier to prevent the RSF rotational angles from changing.

The dependence of the bandgaps of supported bilayer MoS_2_ with RSFs, which were lying on silica substrates, on interlayer distances has been studied^34^. We also plotted the calculated bandgaps and offset crystal momentums of the suspended bilayer MoS_2_ with RSFs on their interlayer distances and found that the dependences were random (see Supplementary Fig. S6).

In addition, based on the above results with bilayer MoS_2_, we speculate rationally that when a RSF exits in a MoS_2_ with more than 2 layers, the CBM position should also change, because the stacking mode of the component layers of the MoS_2_ also deviate from the standard AB stacking. Then, the values of the bandgap and the offset crystal momentum in the band structure should also change, because they are determined by the positions of CBM and VBM.

## Conclusion

In summary, three RSFs with different rotational angles have been found to coexist in a freestanding bilayer MoS_2_ sheet. Their atomic structures have been directly imaged using an AC TEM and compared with HRTEM simulations. Our analyses indicate that the in-plane rotations in the RSFs were induced by a crack and two dislocations in one monolayer of the single MoS_2_ sheet, where the other monolayer was a defect-free MoS_2_ monolayer. The superposition of the two monolayers to form the single bilayer MoS_2_ sheet gave rise to the Moiré patterns of the RSFs in the HRTEM. First-principles calculations manifest that the electronic structures are affected by these RSFs, resulting in RSF angle (*x*)-dependent changes in the bandgap (*E*_g_) and offset crystal momentum (*p*). The changes reveal symmetries about the RSF angle of 30°, *E*_g_ (*x*) ≈ *E*_g_ (60° − *x*) and *p* (*x*) = *p* (60° − *x*), due to similarities in atomic structures for specific pairs of RSF angles. Our work shows that by engineering specific RSFs into 2D MoS_2_ bilayers, the MoS_2_ electronic structures can be tailored to meet the specifications of particular devices for electronics, optoelectronics, and photovoltaics.

## Electronic supplementary material

Supplementary Information
