# Supplementary material for: Direct observation of multiple rotational stacking faults coexisting in freestanding bilayer MoS2
Source: Sci Rep. 2017 Aug 16;7:8323. doi: 10.1038/s41598-017-07615-9 (PMC5559605; doi:10.1038/s41598-017-07615-9)
Supplement: Supplementary file 1 — Supplementary Information [file 41598_2017_7615_MOESM1_ESM.pdf]

Supplementary Information for

Direct observation of multiple rotational stacking faults coexisting in freestanding bilayer MoS_2

Zuocheng Li[1], Xingxu Yan[1], Zhenkun Tang[2,3], Ziyang Huo[4], Guoliang Li[5], Liying Jiao[6], Li-Min Liu[2,*], Miao Zhang[7,*], Jun Luo[1,5,*] & Jing Zhu[1,*]

[1]National Center for Electron Microscopy in Beijing, School of Materials Science and Engineering, The State Key Laboratory of New Ceramics and Fine Processing, Key Laboratory of Advanced Materials (MOE), Tsinghua University, Beijing 100084, China

[2]Beijing Computational Science Research Center, Beijing 100094, China

[3]College of Physics and Electronics Engineering, Hengyang Normal University, Hengyang 421008, China

[4]Queensland Micro- and Nano Centre, Griffith University, Brisbane 4111, Australia

[5]Center for Electron Microscopy, TUT-FEI Joint Laboratory, Institute for New Energy Materials & Low-Carbon Technologies, School of Materials Science and Engineering, Tianjin University of Technology, Tianjin 300384, China

[6]Key Laboratory of Organic Optoelectronics & Molecular Engineering, Department of Chemistry, Tsinghua University, Beijing 100084, China

[7]Chemical Sciences Division, Lawrence Berkeley National Laboratory, Berkeley, California 94720, USA

Table of Contents

1. Methods (Page 3)

2. Supplementary figures (Pages 4-9)

1. Methods

AC TEM. The spherical aberration was set to be negative, and the corresponding twofold astigmatism A_1, threefold astigmatism A_2, and coma B_2 were always less than 2 nm, 100 nm, and 40 nm, respectively, when the HRTEM images were taken. These conditions are similar to those reported.[40] All TEM experiments were performed under the electron beam energy of 80 kV. The beam intensity (dose) was 25,600 e $Å^{-2}$ s^{-1}. Under the conditions, the RSFs in Fig. 2a of the main text formed after a beam exposure of 317 s in duration.

First-principles calculations. The calculations were performed using the Vienna Ab Initio Simulation Package (VASP).[42–44] The generalized gradient approximation (GGA) with the Perdew, Burke and Ernzerhof (PBE) functional[45] was used to treat the exchange-correlation interaction between electrons, and the electron−ion interactions were treated in projector augmented wave (PAW) formalism.[46] A vacuum region larger than 15 Å and perpendicular to the MoS_2 sheets (along the c axis) was applied to avoid the interaction between the sheets in neighboring cells caused by the periodic boundary condition. In our calculation, a kinetic-energy cutoff for plane-wave expansion was set to 500 eV. All atoms in each unit cell were fully relaxed until the force on each atom was less than 0.005 eV/Å. Electronic energy minimization was performed with a tolerance of 10^{-6} eV. The DFT-D3 approach[47] was used in order to take into account the effect of the van der Waals interaction.

2. Supplementary figures

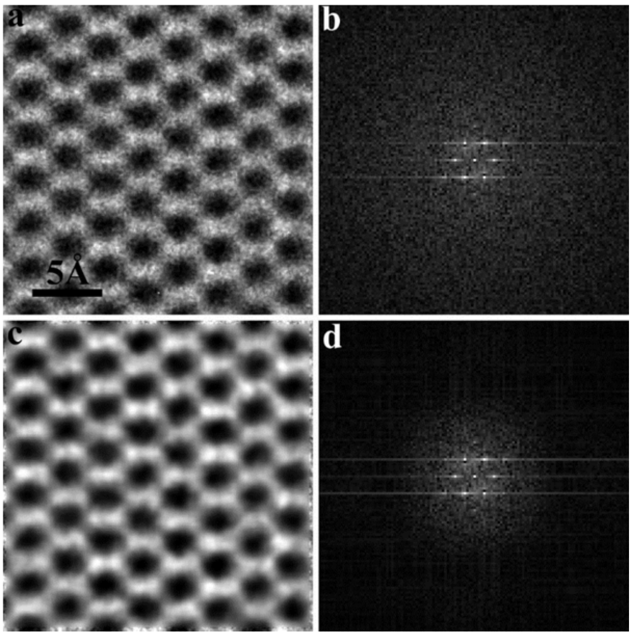

Figure S1. (a, b) Raw HRTEM image and its FFT pattern of Fig. 1c of the main text. (c, d) Filtered image of (a) and its FFT pattern, which are the same as Fig. 1c, d of the main text, respectively. The filtering is low-pass and has been widely used to remove noise and increase visibility of HRTEM images.[23–32] All of the spots in (b) are contained in (d), indicating that the structural information in (a) is properly contained in (c) and no artifacts exist.[23–32]

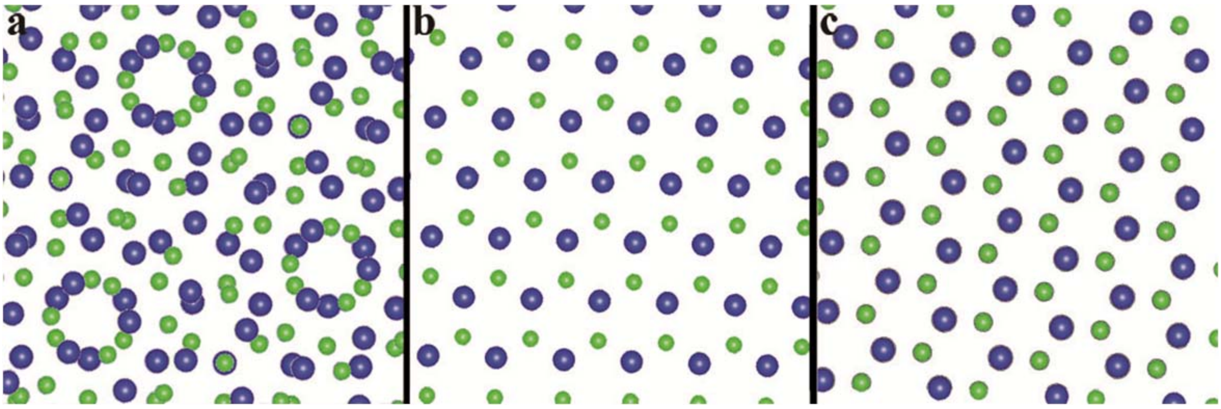

Figure S2. (a) Top view of the atomic model of Fig. 3a, where the blue and the green dots denote the Mo and S atoms, respectively. (b, c) Top views of the two component layers of the model. The model in (a) was constructed by superposing the two component layers in (b) and (c), where the angle between the orientations of (b) and (c) was 27.80º and their interlayer spacing was 6.5 Å. The value of 6.5 Å is standard for 2D MoS_2.[1,2]

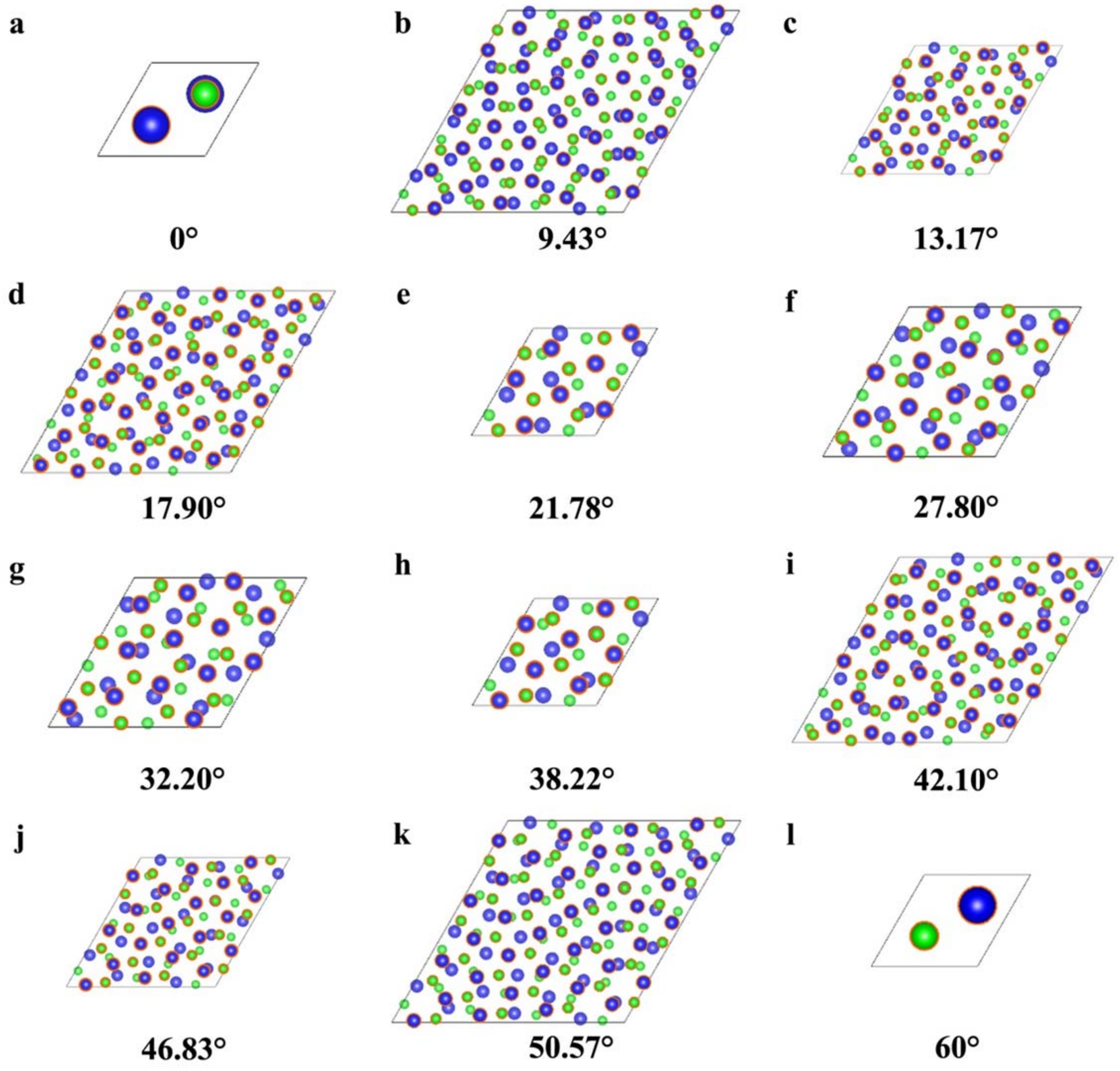

Figure S3. Top views of the unit cells of bilayer MoS_2 with and without RSFs, where the values of the rotational angles are labeled and the atoms of the upper and the lower layers of each unit are drawn to be with and without orange circles, respectively, to make them distinguishable. (a) and (l) are the unit cells of the AB- and the AA-stacked bilayer MoS_2, respectively, which can be considered to contain the RSFs with the rotational angles of 0º and 60º, respectively.

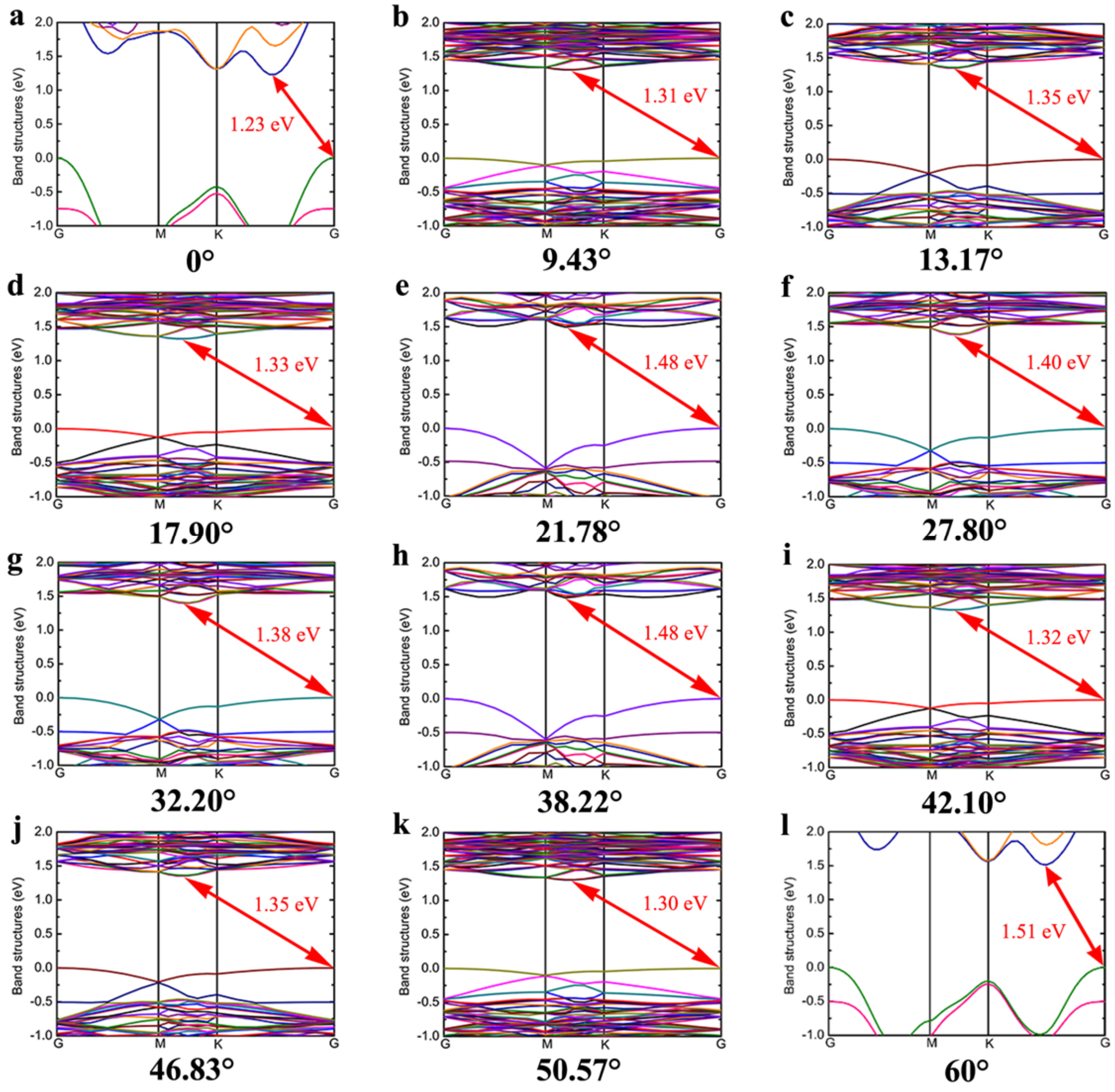

Figure S4. Calculated electronic structures of the materials in Fig. S3. The two ends of each arrow indicate the positions of CBM and VBM. The red numbers with the unit of eV denote the indirect bandgap values.

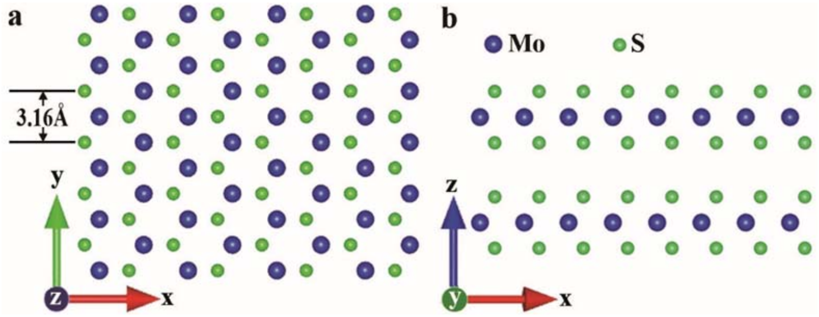

Figure S5. Atomic structure of the AA-stacked bilayer MoS_2. (a, b) Top and side views of the atomic model of the AA-stacked MoS_2.

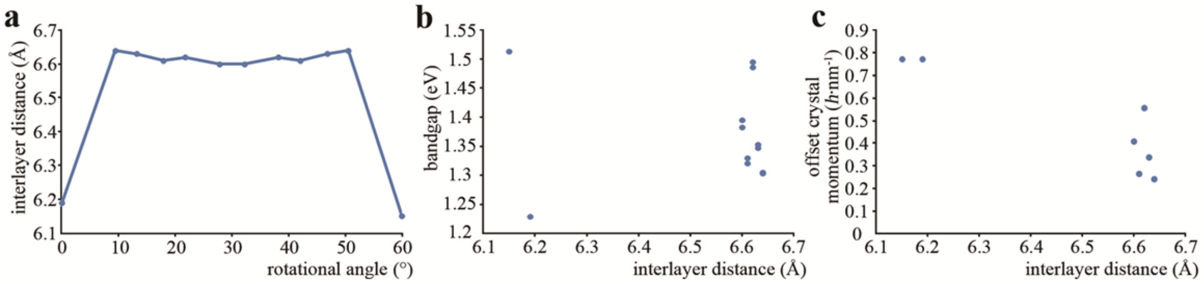

Figure S6. Calculated interlayer distances (a) of the unit cells of bilayer MoS_2 without and with RSFs and the dependences of the indirect bandgaps (b) and the offset crystal momentums (c) on the distances. The unit cells with RSFs of 0º and 60º correspond to the AB- and the AA-stacked bilayer MoS_2, respectively. The values of the interlayer distances of the unit cells in (a) are nearly symmetrical about the rotational angle of 30°, reflecting the symmetry of the unit cells. The average of the interlayer distances is 6.545 Å, close to the standard value (6.5 Å) of 2D MoS_2.[1,2]
